# Alternative splicing of human peroxisome proliferator-activated receptor delta (PPARdelta):effects on translation efficiency and *trans*-activation ability

**DOI:** 10.1186/1471-2199-8-70

**Published:** 2007-08-16

**Authors:** Kerstin Lundell, Petra Thulin, Anders Hamsten, Ewa Ehrenborg

**Affiliations:** 1 The Atherosclerosis Research Unit, King Gustaf V Research Institute, Department of Medicine, Karolinska Institutet, Stockholm, Sweden

## Abstract

**Background:**

Peroxisome proliferator-activated receptor delta (PPARδ) is a member of the nuclear receptor superfamily. Numerous studies have aimed at unravelling the physiological role of PPARδ as a transcriptional regulator whereas the regulation of PPARδ gene expression has been less studied.

**Results:**

The principal transcription start site in the human PPARδ gene identified here is positioned upstream of exon 1, although four alternative 5'-ends related to downstream exons were identified. The demonstration of multiple 5'-UTR splice variants of PPARδ mRNA, with an impact on translation efficiency, suggests a translational regulation of human PPARδ expression. Five untranslated exons identified in this study contribute to the variability among the 5'-UTRs of human PPARδ mRNAs. Moreover, *in vitro *studies of a 3'-splice transcript encoding a truncated variant of PPARδ (designated PPARδ2) show that this isoform constitutes a potential dominant negative form of the receptor.

**Conclusion:**

We propose that alternative splicing of human PPARδ constitutes an intrinsic role for the regulation of PPARδ expression and thus activity, and highlight the significance of alternative splicing of this nuclear receptor in physiology and disease.

## Background

Peroxisome proliferator-activated receptors (PPARs) are ligand-dependent transcription factors that activate target genes by binding to peroxisome proliferator response elements (PPREs) as heterodimers with the retinoid × receptor (RXR) (NR2B). Three PPAR isoforms encoded by different genes have been identified: α (PPARα or NR1C1), δ/β (PPARδ or NR1C2) and γ (PPARγ or NR1C3). The functional specificity among members of the PPAR subfamily is achieved by isoform-specific tissue expression as well as ligand binding specificity [1-5]. Endogenous ligands that activate PPARs are polyunsaturated fatty acids or their derivatives [6-8]. Ligand activation of PPARs induces conformational changes that promote binding of co-activators that are essential for the *trans*-activating function whereas binding of nuclear co-repressors is associated with transcriptional repression. PPARδ is the only isoform that maintains repressor activity when bound to DNA. It has been reported that unligated PPARδ can act as an intrinsic transcription repressor and inhibit the *trans*-activation activity of other PPARs [9-12]. Given that the PPARδ gene is ubiquitously expressed this suggests an additional role for PPARδ as a receptor whose relative level of expression can be used to repress the expression of PPRE target genes.

It was not until recently that the roles of PPARδ were disclosed through utilization of specific ligands together with relevant cells and animal models. PPARδ controls various physiological functions including lipid and glucose homeostasis, inflammation, cell proliferation and differentiation [13]. PPARδ-deficiency in mice negatively affects growth, adipose stores, epidermal cell proliferation, and myelinisation of corpus callosum [14,15]. Treatment of insulin-resistant obese rhesus monkeys with a PPARδ specific ligand has been shown to promote cholesterol efflux, increase HDL levels and decrease LDL, triglyceride and insulin levels [16]. Further, PPARδ is highly expressed in skeletal muscle, and treatment with PPARδ specific ligands increases the proportion of oxidative slow-twitch myofibers and induces expression of genes involved in fatty acid utilization and oxidation as well as glucose uptake and metabolism [17-24].

Little is so far known considering how the expression of PPARδ itself is regulated. Here we report on new 5'-untranslated exons and multiple 5'-UTRs of PPARδ mRNA transcripts, with an impact on the translation efficiency. In addition, we report on the expression of a 3'-splice variant of human PPARδ (PPARδ2), encoding a potential dominant negative regulator of gene expression. Since alternative splicing contributes to the regulation of gene expression and constitutes an important source of evolutionary diversity, we hypothesize that spliced mRNA isoforms of human PPARδ play a role in controlling its function.

## Results

### Multiple untranslated exons and alternative transcription start sites

**Figure 1:**
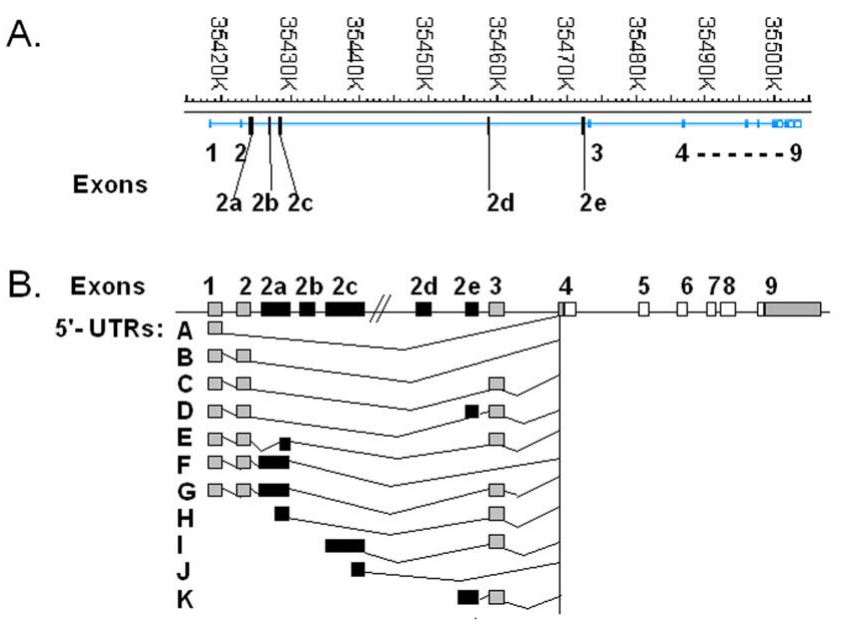
**Structure and chromosomal location of the human PPARδ gene**. *A*. A physical map of the 85 kb human 6p21.31 region. The locations of exons are outlined with the previously known exons (denoted in *upper *row) and newly identified exons (denoted in *lower *row) indicated. The genomic positions of the new exons were deduced by comparing their sequences to that of the human PPARδ gene [GenBank: NM_006238]. The sequences of splice junctions and alternative 5'-ends related to these exons are outlined in Tables 1 and 2, respectively. *B*. A schematic representation of the PPARδ gene showing coding exons (*white *boxes), previously reported untranslated exons or part of exons (*grey *boxes), and herein identified untranslated exons (*black *boxes). The analysed and discussed variety of splicing among untranslated exons and alternative 5'-ends identified by Marathon 5'-RACE as described in "Methods" is shown below the gene (*5'-UTRs: A-K*).

We have previously reported the human PPARδ gene to encompass 9 exons of which exons 1–3, the 5'-end of exon 4 and the 3'-end of exon 9 are untranslated [25]. An investigation was conducted aiming to identify possible alternative 5'-ends in transcripts of PPARδ. Five new 5'-untranslated exons in alternatively spliced transcripts of human PPARδ mRNA were identified. The locations of these exons (designated exons 2a – 2e) in the gene sequence are outlined in Figure 1A. The newly identified exons were found at different time-points during the investigation and were subsequently re-annotated to indicate the relative position of each exon in the gene. Exons 2a, 2c and 2e were originally found by 5'-RACE using Marathon cDNAs from placenta, adipose tissue and pancreas. Combinations of these exons, including differently spliced forms of exon 2a, were found to be spliced-in generating a variety of 5'-UTRs, as displayed in Figure 1B. Exons 2b and 2d were subsequently found among sequenced PCR fragments generated using Marathon cDNAs and cell-derived cDNAs as templates and different combinations of 5'-untranslated exon-specific primers. The genomic locations and sequences of the splice junctions of spliced-in exons are summarized in Table 1. None of the 5'-UTRs found in this study contains any new in-frame coding regions. Thus, the open reading frame (ORF) region encoded by exons 4 to 9 remains identical in all 5'-spliced transcripts.

**Table 1:** Sequences of splice junctions of newly identified spliced-in exons in the 5'-UTR of human PPARδ

**Spliced-in exon**	**Size (bp)**	**Genomic position** ** downstream of exon 2 (bp)**	**3'-splice acceptor**	**5'-splice donor**
2a	641	1212	ctgc**ag**GGGTAG	TCGCAG**gt**agga
2b	119	4427	agat**ag**CATCTC	CTACAG**gt**atgt
2c	122	5778	tctc**ag**GACCAG	TGTGAG**gt**aatg
2d	315	36426	atct**ag**GAGGTG	TGGGAA**gt**gagg
2e	115	49826	tttc**ag**ATTATC	TGTCCT**gt**gagt

The genomic positions of exons were deduced using the human PPARδ RefSeq [GenBank: NM_006238]. Upper-case and lower-case letters indicate exon and intron sequences, respectively. The consensus splice donor-acceptor sequences are in bold letters.

The majority of the 5'-splice PPARδ transcripts contain exon 1 in the 5'-end. However, alternative 5'-ends associated with exons 2a, 2c and 2e were found, which is illustrated in Figure 1B, *5'-UTRs: H-K*. Of these, the alternative 5'-end containing full-length exon 2c was found only in pancreas (Figure 1B, *5'-UTR: I*) whereas the others could be found in all three Marathon cDNAs. The genomic locations and sequences of the alternative 5'-end exons are summarized in Table 2.

**Table 2:** Sequences of identified alternative 5'-end exons in 5'-UTRs of human PPARδ transcripts

**5'-end exon**	**Size (bp)**	**Genomic position ** **downstream of exon 2 (bp)**	**5'-end**	**5'-splice donor**
2a	79	1774	CCCAGTGGCAGC	TCGCAG**gt**agga
2c	883	5017	GCCAGTTCTTTT	TGTGAG**gt**aatg
2c	318	5582	CTTGATGCTGTT	TGTGAG**gt**aatg
2e	180	49761	CAACCTCCCTGC	TGTCCT**gt**gagt

The genomic positions of exons were deduced using the human PPARδ RefSeq [GenBank: NM_006238]. Upper-case and lower-case letters indicate exon and intron sequences, respectively. The consensus splice donor sequences are in bold letters. The relative positions of 5'-ends in the gene are also illustrated in Figure 1*B*, 5'-UTRs denoted H-K.

A bioinformatic approach to identify splice variants of human PPARδ using the NCBI human Genome Browser and the human EST-search tool, indeed, showed the presence of exons 2a, 2b, 2c and 2d among 5'-spliced transcripts of PPARδ, all with exons 1 and/or 2 upstream.

### Expression of PPARδ 5'-UTR isoforms in human cell lines and tissues

**Figure 2:**
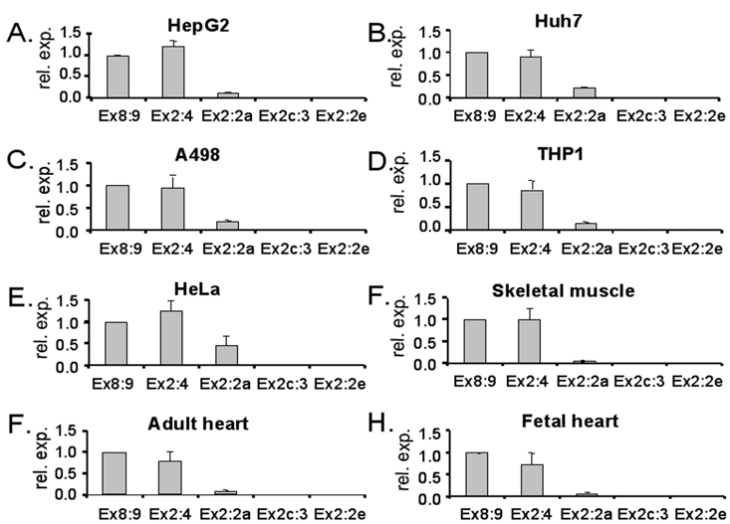
**TaqMan analysis of splice products in human cell lines and tissues**. Real-time PCR on cDNA from human cell lines (A-E) and human tissues (F-H) using primers and probes specified in Table 3 was performed as described in "Methods". The following combinations of primers and probes were used for each TaqMan assay; TQEx8Fw, TQEx9Rev and Ex8:9Pr (Ex8:9); TQEx2Fw, TQEx4Rev and Ex2:4Pr (Ex2:4); TQEx2Fw, TQEx2aRev and Ex2:2aPr (Ex2:2a); TQEx2cFw, TQEx3Rev and Ex2c:3Pr (Ex2c:3); TQEx2Fw, TQEx2eRev and Ex2:2ePr (Ex2:2e). The result of each assay was normalized to the result of the Ex8:9 assay using the comparative C_T _method and presented as the mean relative expression levels (± SD).

Total RNA extracted from five different human cell lines was analysed by RT-PCR using different combinations of exon-specific PPARδ primers. The presence of PPARδ transcripts containing exons 2a, 2c and 2e in all cell lines was confirmed by sequencing of PCR products (results not shown). Real-time PCR was subsequently conducted to estimate the relative amount of isoforms containing exons 2a, 2c and 2e among transcripts of PPARδ mRNAs. Samples of cDNA from human cell lines and from human skeletal muscle tissue as well as from adult and fetal heart tissues were subjected to TaqMan analysis using primers and probes specified in Table 3. The analysis, illustrated in Figure 2, showed that in all cell lines and tissues studied the most common splice variant contained exons 2 and 4 joined together (Ex2:4) in the 5'-UTR. This variant was expressed in the same order of magnitude as the total amount of full length PPARδ detected with primers targeting exons 8 and 9 (Ex8:9). Transcripts containing exon 2 joined to exon 2a (Ex2:2a) were expressed at a much lower level in all cell lines and tissues even though the level of expression varied and was higher in HeLa cells compared to skeletal muscle. The expression levels of transcripts containing isoforms joining exons 2c and 3 (Ex2c:3) as well as exons 2 and 2e (Ex2:2e) were below the detection limit in all the cell lines and tissues studied.

**Table 3:** TaqMan primers and probes used in this study

**Name**	**Target**	**Sequence (5'-3')**
Forward primers		
TQEx2Fw	exon 2	5'-GCTCACCAACAGATGAAGAC
TQEx2cFw	exon 2c	5'-CCTCCTTGGAGACCAGCTA
TQEx8Fw	exon 8	5'-GACCTGGCCCTATTCATTG
Reverse primers		
TQEx2aRev	exon 2a	5'-GCATTGTTCAGACTCTTGGT
TQEx2eRev	exon 2e	5'-CAGGGAAGGTTCAAGGTCAA
TQEx3Rev	exon 3	5'-GTATCTGACCCTGCTTTCCA
TQEx4Rev	exon 4	5'-TCTGAACGCAGATGGACCT
TQEx9Rev	exon 9	5'-ACCCGTGGAACGTTCATGA
Probes		
Ex2:2aPr	Exons 2:2a	5'-FAM-ACCTCCTACCCCTCGTTGGTG-TAMRA
Ex2:2ePr	Exons 2:2e	5'-FAM-CACCAACGAGATTATCTTGAAGAC-TAMRA
Ex2c:3Pr	Exons 2c:3	5'-FAM-CATTCCAGACCCTCACATAAGGA-TAMRA
Ex2:4Pr	Exons 2:4	5'-FAM-TCCCATCAGCCTCGTTGGTGC-TAMRA
Ex8:9Pr	Exons 8:9	5'-FAM-CTGGCCGGTCTCCACACAGAA-TAMRA

The primers and probes are annotated according to the target exons and exon/exon junctions in the human PPARδ gene, respectively.

### Analysis of promoter activities

**Figure 3:**
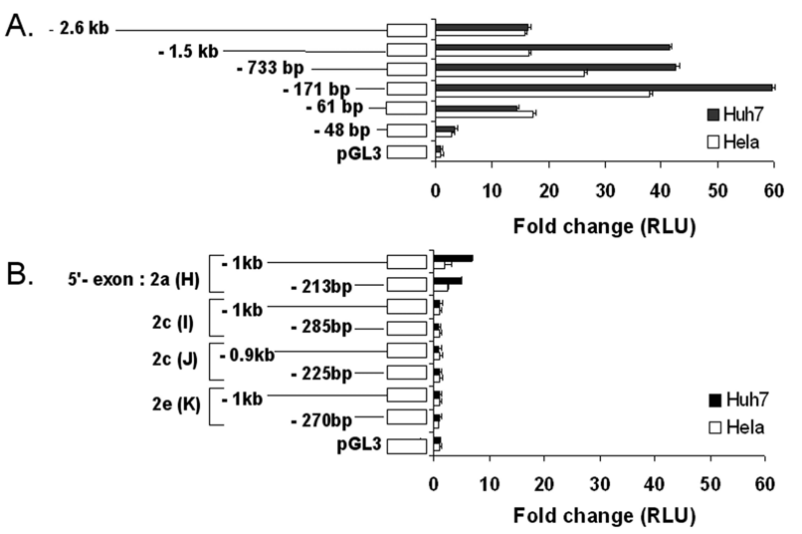
**Reporter gene constructs and relative promoter activities**. A schematic view of the different PPARδ reporter gene constructs and the luciferase activities obtained after transient transfection in Huh7 (*black *bars) and HeLa cells (*white *bars), setting the value of the promoterless control plasmid to 1. *A*. Luciferase assays on reporter gene constructs containing from 2.6 kb down to 48 bp of the region upstream of exon 1 in the PPARδ gene compared to empty pGL3 vector. *B*. Luciferase assay on reporter gene constructs containing approximately 1 kb and 250 bp of the upstream regions of four identified alternative 5'-ends summarized in Table 2 and illustrated in Figure 1*B *(*5'-UTRs: H-K*), compared to empty pGL3 vector.

The majority of the 5'-splice PPARδ transcripts identified contain exon 1 in the 5'-end, indicating that the previously identified promoter region upstream of exon 1 is the major region for transcriptional regulation of the human PPARδ gene [25]. This was confirmed by transient transfection assays in both Huh7 and HeLa cells using a range of reporter gene constructs containing 2.6 kb to 48 bp of the DNA sequence upstream of exon 1 (Figure 3). Maximum luciferase activity was obtained with the reporter construct containing 171 bp of the promoter in both cell lines (Figure 3A). Shorter constructs showed declining activities with a major drop using the 48 bp construct. Likewise, longer constructs showed decreasing luciferase activities, indicating the presence of upstream transcriptional repressor element(s).

Reporter gene constructs encompassing approximately 1 kb and 250 bp, respectively, of the 5'-regions of each of the four putative transcription start sites (summarized in Table 2 and illustrated in Figure 1B, *5'-UTR: H-K*) showed no or weak basal promoter activity in Huh7 and Hela cells compared with the identified promoter region upstream of exon 1 (Figure 3B). *In silico *analysis revealed a potential PPRE located -86 to -74 bp upstream of the identified alternative 5'-end within exon 2a. However, the two reporter gene constructs (1 kb and 213 bp) containing this PPRE were neither affected by overexpression of PPARδ nor PPARα combined with isoform-specific ligands GW501516 [26] and fibrates, respectively (results not shown).

### Translation efficiency of PPARδ 5'-splice variants

**Figure 4:**
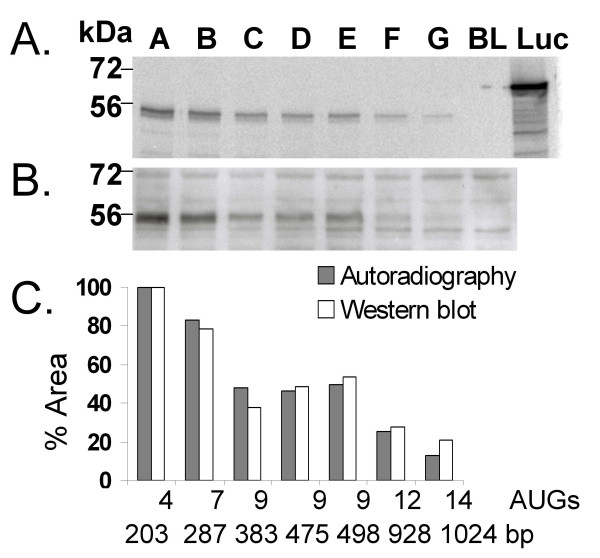
**The compiled results of the *in vitro *coupled transcription/translation analysis**. *A*. Denaturing gel analysis of [^35^S]methionine labelled translation products visualized by autoradiography. Samples A-G correspond to the *in vitro*-translated products obtained using Quick CoupledTranscription/Translation System (Promega) as described in "Methods" with plasmid DNA templates containing human PPARδ with the 5'-UTRs denoted A-G in Figure 1*B*. The composition of untranslated exons in the 5'-UTRs are: (A) Exon 1, (B) Exons 1+2, (C) Exons 1+2+3, (D) Exons 1+2+2e+3, (E) Exons 1+2+spliced 2a(90 bp)+3, (F) Exons 1+2+2a, (G) Exons 1+2+2a+3. BL is a negative control sample without template and Luc is a positive luciferase control sample. *B*. Western blot analysis of the products of the translation reactions probed with the polyclonal PPARδ specific antibody IMG-3297. The sizes of standard molecule markers are given on the left side (*A *and *B*). *C*. Quantification of the [^35^S]methionine labelled band intensities was performed on the autoradiographic (grey bars) and Western blot (white bars) bands using the NIH ImageJ software. The relative intensities of signals (% Area) were calculated setting the signal of construct A to 100%. The number of upstream AUGs and the total length of each 5'-UTR is given below each bar of calculated signal.

The impact of different 5'-UTRs on the translation efficiency was analysed using coupled transcription/translation in a reticulocyte system (Promega). Plasmids harbouring seven different 5'-UTRs (denoted *5'-UTR: A-G *in Figure 1B) placed in front of the PPARδ coding region were analysed. The compiled results outlined in Figure 4 clearly show that transcripts with extended 5'-UTRs are less efficiently translated into protein. The [^35^S]methionine labelled translation products were resolved by SDS-PAGE and subjected to autoradiography (Figure 4A). Western blot analysis of the translated protein (Figure 4B), using a PPARδ specific antibody, showed immunoreactive proteins of compatible sizes (approximately 50 kDa) and intensities as the autoradiogram (Figure 4A). The intensities of the [^35^S]methionine and Western blot bands were quantified (Figure 4C). The alternatively spliced human PPARδ 5'-UTRs differ in length and number of AUGs. All identified upstream AUGs are upstream open reading frames (uORFs) with an in-frame stop codon within the 5'-UTRs. The 5'-UTR sequences analysed harbour from 4 up to 14 upstream AUGs (Figure 4C). Strong inverse relationships were observed between the number of AUGs and the measure of quantified translation efficiency (% Area) (R = 0.97 autoradiography; R = 0.90 Western blot) whereas that between the 5'-UTR length and % Area was less pronounced (R = 0.84 autoradiography; R = 0.71 Western blot).

### Characterization of the truncated human PPARδ isoform (PPARδ2)

A 3'-spliced isoform of human PPARδ, formed by exon 9 skipping, has been reported from human placenta cDNA clones [EMBL: BC007578 and BC002715, RefSeq GenBank: NM_177435]. Intron 8 is retained in the primary transcript and an in-frame stop codon (UGA) in the extended exon 8 encodes a protein lacking the C-terminal 82 amino acids of PPARδ, which constitutes the end of the ligand-binding domain. The presence of a PPARδ2 transcript (identical to EMBL:BC007578 and BC002715) with a polyadenylation signal 583 nt downstream of the stop codon and a poly(A)tail attached was confirmed by 3'-RACE using cDNA from placenta and adipose tissue (data not shown). Adaptor linked transcripts with a 3'-end within intron 8 were also obtained from pancreas cDNA, however, poly(A)tail linked 3'-end transcripts could not be confirmed.

**Figure 5:**
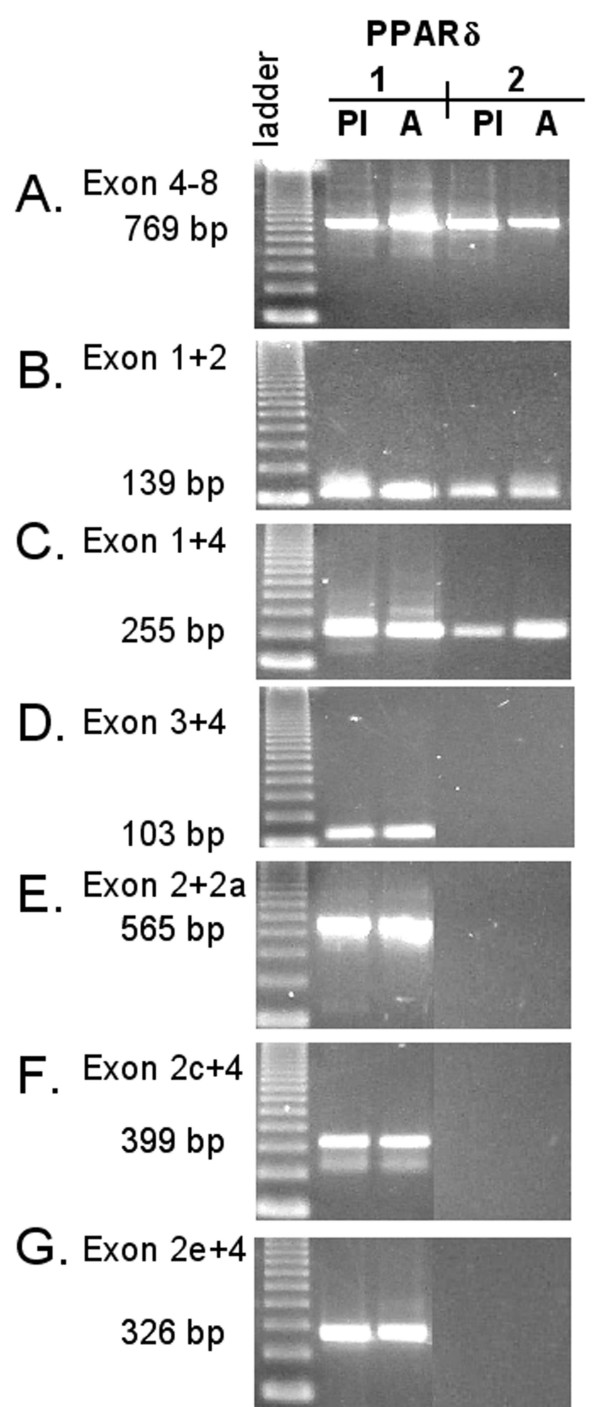
**PPARδ isoform-specific variability among 5'-UTRs**. 5'-RACE products enriched in either full-length PPARδ (PPARδ1) or PPARδ2 obtained using cDNA from placenta (Pl) and adipose tissue (A) according to "Methods" were used as templates for PCR reactions with untranslated exon/exon primer pairs, and with primers that amplify over exons in the coding region as control. *A*. Exon 4 to 8 (769 bp) amplified using Ex4Fw and Ex8Rev; *B*. Exon 1 + 2 (139 bp) amplified using Ex1:2Fw and Ex2Rev; *C*. Exon 1 + 4 (255 bp) amplified using Ex1:2Fw and Ex4:2Rev; *D*, Exon 3 + 4 (103 bp) amplified using Ex3Fw and Ex4:2Rev; *E*, Exon 2 + 2a (565 bp) amplified using Ex2Fw and Ex2a:1Rev;*F*, Exon 2c + 4 (399 bp) amplified using Ex2cFw and Ex4:1Rev;*G*, Exon 2e + 4 (326 bp) amplified using Ex2eFw and Ex4:2Rev. The sizes of the major bands obtained are indicated and the identities of the products were confirmed by sequencing.

PCR amplification of placenta and adipose 5'-RACE products enriched in either full-length PPARδ (PPARδ1) or PPARδ2, respectively, revealed some differences regarding the variety of untranslated exons among 5'-UTRs, as illustrated in Figure 5. A sequence within the coding region (exons 4 to 8) as well as sequences encompassing exons 1 to 2 and exons 2 to 4 could be amplified using all 5'-RACE products (Figure 5A–C). Transcripts containing exons 3, 2a, 2c, and 2e (Figure 5D–G) appeared, on the other hand, to be absent in samples enriched in PPARδ2.

**Figure 6:**
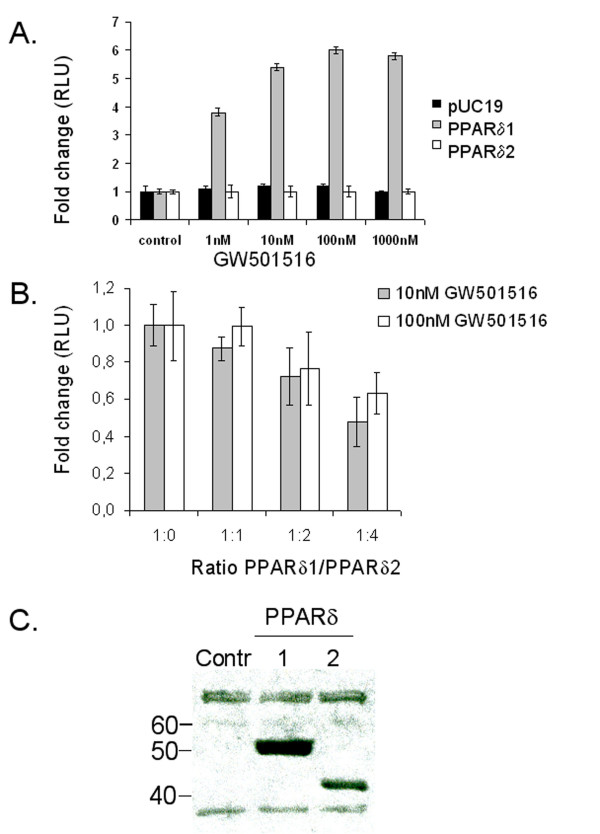
***Trans*-activation ability of PPARδ2 investigated by transient transfection of HeLa cells**. *A*. Co-transfection of pFABPLuc reporter construct (200 ng) with expression vector for either PPARδ1 (100 ng), PPARδ2 (100 ng) or irrelevant plasmid DNA pUC19 (100 ng), untreated (control) or treated with 1 nM, 10 nM or 100 nM of the PPARδ specific ligand GW501516. *B*. Co-transfection of pFABLuc reporter construct (200 ng) with constant amount of expression vector for PPARδ1 (50 ng) and increasing concentrations of the expression vector for PPARδ2 (50 to 200 ng) and treatment with 10 nM or 100 nM GW501516. Transfection of the pFABLuc vector alone was used as a control. Plasmid DNA pUC19 was added to ensure equal amount of DNA in all transfections. Plasmid pSV-β-galactosidase control vector (Promega) (260 ng) was co-transfected in all experiments for normalization of the transfection efficiency. The data presented are the mean (± SD) luciferase/β-galactosidase ratios of three independent transfections determined in quadruplicates. The activities are expressed as relative values setting the value of untreated control to 1 (*A*) or the value obtained without PPARδ2 expression plasmid to 1 (*B*). C. Western blot analysis with nuclear extracts prepared from untransfected HeLa cells (Contr) and HeLa cells transfected with the expression vectors encoding PPARδ1 or PPARδ2, respectively, using a PPARδ antibody raised against the N-terminal region of the nuclear receptor (sc-7197, Santa Cruz Biotechnology). The sizes of standard molecule markers are given on the left side.

**Figure 7:**
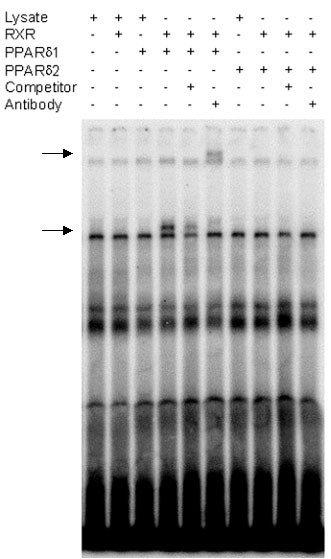
**EMSA to assess the binding properties of PPARδ isoform 1 and 2 to ACO-PPRE**. Gel retardation assay was performed with end-labelled ACO-PPRE in the presence (+) or absence (-) of *in vitro *transcribed/translated human PPARδ1, PPARδ2, RXRα or mock lysate as described in "Methods". Unlabelled ACO-PPRE was added at 100-fold molar excess for competition. Supershift experiments were carried out using a PPARδ antibody directed against the N-terminal region of human PPARδ. *Arrows *indicate the positions of the shifted and the supershifted bands. None of the receptors (PPARδ1, PPARδ2 or RXRα) alone could be supershifted in the presence of the PPARδ antibody (data not shown).

To analyse the *trans*-activating ability of PPARδ2, the pFABPLUC reporter activity was investigated during overexpression of PPARδ2 in the absence or presence of the expression vector for full-length PPARδ (PPARδ1) and the PPARδ specific ligand GW501516 [26]. Co-transfection of pFABPLuc and the expression vector for PPARδ1 in HeLa cells and treatment with GW501516 (10 nM and 100 nM) increased the *trans*-activation ability in a dose-dependent manner (Figure 6A). PPARδ2, on the other hand, had no *trans*-activation ability in the presence of GW501516 (Figure 6A) but rather repressed the ligand-induced activation of pFABPLuc reporter by PPARδ1 (Figure 6B). The expression of PPARδ1 and PPARδ2, respectively, in transfected HeLa cells was confirmed by Western blot analysis showing immunoreactive bands of expected sizes (approximately 50 kDa and 40 kDa) (Figure 6C). Gel retardation analysis showed that full-length PPARδ1 binds to the functional PPRE of the rat acyl-CoA oxidase gene in the presence of RXR whereas PPARδ2 does not bind (Figure 7).

### Comparative genomics

**Figure 8:**
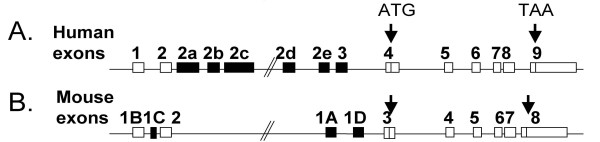
**Comparing the organization of the human and mouse PPARδ genes**. *A*. The human PPARδ gene (85 kb) located on chr6, *B*. The mouse ortologue gene (70 kb) located on chr17. The overall difference in length can be accounted for by the 63 kb versus 50 kb separating exon 2 and the first coding exon (4 or 3) in human and mouse genes, respectively. The relative positions of exons along the genes are given, specifying exons that are conserved between species (*white *boxes) and species-specific exons (*black *boxes). *Arrows *indicate the position of the initiation codon ATG and the stop codon TAA, respectively, with the intervening coding exons.

**Figure 9:**
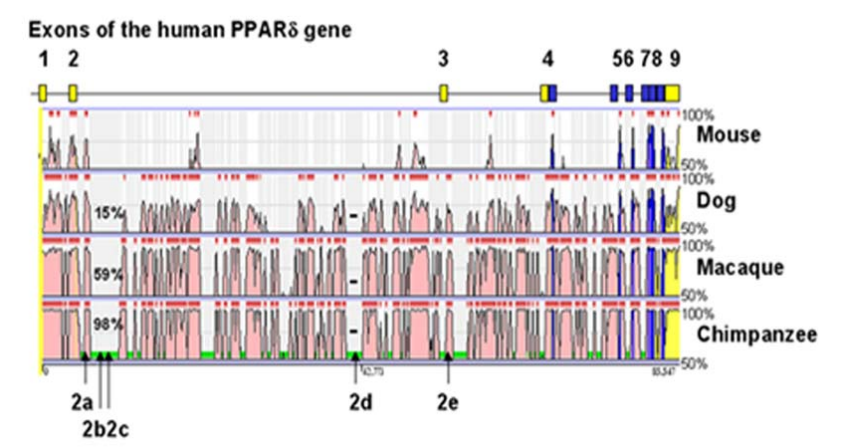
**Conservation profiles of the PPARδ gene using the ECR Browser tool**. The conservation profiles (percent identity cut-off of 50% to 100%) of the human PPARδ gene in comparison with the mouse (*Mus musculus*; chr17), dog (*Canis familiaris; *chr12), macaque (*Macaca mulatta*; chr4) and chimpanzee (*Pan troglodytes*; chr6) orthologous genes are shown. Conserved sequences are defined as coding exons (blue), untranslated exons (yellow) and introns (pink). The locations of the novel untranslated human exons (2a-2e) in the PPARδ gene are indicated by arrows. The percent identity of the masked (unaligned) sequence harbouring exons 2b and 2c obtained by ClustalW alignment is indicated in the defined area, likewise the lack of identity over the masked region containing exon 2d is indicated by horizontal lines. A schematic view showing the locations of previously identified exons in human PPARδ gene are aligned above the conservation profile for orientation.

The human and mouse PPARδ RefSeqs were compared and revealed an overall conserved pattern of exons and introns. Two 5'-untranslated exons are conserved between the human and mouse genes as illustrated in Figure 8. The mouse exon 1A, previously reported to be positioned upstream of exon 1B [27], was found to be located downstream of exon 2 in the mouse RefSeq. The two genes harbour six and three, respectively, species-specific 5'-untranslated exons. All these exons, except for mouse exon 1C, are located between exon 2 and the first coding exon, a region with no apparent sequence conservation between species. Comparative analysis using the ECR Browser tool [28], on the other hand, revealed regions of conservation relative to the human PPARδ gene in the chimpanzee, rhesus macaque and dog orthologous genes as depicted in Figure 9. The genomic positions of the human PPARδ exons 2a and 2e within evolutionary conserved regions and of exons 2b, 2c and 2d within repetitive regions are indicated in Figure 9. Repetitive regions are masked prior to comparative analysis of conserved region in the ECR Browser alignment and thus excluded from comparison. Using ClustalW alignment the masked regions harbouring the sequences of the orthologous exons 2b and 2c in chimpanzee, macaque and dog revealed an overall sequence identity of 98%, 59% and 15%, respectively, compared with the human PPARδ counterpart, as indicated in Figure 9. The masked region containing exon 2d, on the other hand, showed no sequence similarity between species. Identity scores and lengths of matching sequences to the human exons identified in the PPARδ gene of chimpanzee, macaque and dog are given in Table 4.

**Table 4:** Sequence identity scores of human PPARδ exons in comparable regions of orthologous genes

**Human (bp)**	**Chimpanzee Score (bp)**	**Macaque Score (bp)**	**Dog Score (bp)**
Exon 2 (84)	98 (84)	97 (84)	85 (84)
Exon 2a (641)	98 (641)	95 (639)	*82 *(622)†
Exon 2b (119)	99 (119)	74 (122)	-
Exon 2c (883)	98 (852)	-*	*77 *(507)†
Exon 2d (312)	-	-	-
Exon 2e (115)	99 (129)	*89 *(66)†	*58 *(141)‡
Exon 3 (96)	99 (96)	*95 *(84)†	*39 *(103)‡
Coding exons	99	95	91

Identity scores were calculated by ClustalW alignments and include scores for the novel human PPARδ exons and human exon 2, exon 3 and coding exons (1326 bp) for comparison. Exon 1 was not included since complete sequence information for this exon is not available for all species. Scores given in *italic *numbers indicate a sequence without conserved splice junctions.

* Sequence comparison precluded by unsequenced gaps in the macaque PPARδ gene

†A full-length exon is not conserved. Identity scores are given for a sequence homologous to the 3'-region of the comparable human exon

‡ An orthologous exon was not identified. Identity scores are given for a sequence with the highest homology scores within the genomic region comparable to the location of the human exon

The overall sequence conservation between human and chimpanzee PPARδ genes is pronounced. Moreover, the NCBI HomoloGene database displays two isoforms of chimpanzee PPARδ mRNA, denoted isoforms 3 [GenBank: XM_001172224] and 4 [GenBank: XM_001172238], which harbour spliced forms of exon 2a.

The major process whereby new exons evolve is considered to be exonization of intronic sequences [29,30]. The primate specific Alu-elements can be exonized through a small number of mutations. Using the RepeatMasker to analyse the human PPARδ gene, exon 2b was found to fulfil the criteria of being an Alu-derived exon [31,32]. Similar antisense Alu-elements are present in the corresponding region of the chimpanzee and macaque genes, both with mutations to enable exonization of orthologous exon 2b. A second exon-associated Alu-element is present in the 5'-end of exon 2c, close to the alternative 5'-end identified in pancreas. This Alu-element is conserved in the chimpanzee PPARδ gene. Exon 2d is located within a SVA element (SVA_D), named after its main components; short interspread nuclear element (SINE), variable number of tandem repeats (VNTR) and Alu [33]. In general this subfamily of SVA elements is believed to predate the divergence of human and chimpanzee lineage but is not present in the chimpanzee PPARδ gene. Exon 2d is thus truly a human specific exon.

Homologues to the human PPARδ2 isoform are presented in the NCBI GenBank as isoform models for both chimpanzee and macaque [GenBank: XM_001172194 and XM_001116671]. The stop codon and the polyadenylation signal in intron 8 are fully conserved in the PPARδ genes of these primates. The potential 3'-UTRs of chimpanzee and macaque PPARδ2 show 99% and 94% overall sequence identity to the human counterpart, respectively. This pronounced sequence conservation indicates that PPARδ2 could be a primate specific isoform.

## Discussion

A diversity of 5'-UTRs derived from alternative splicing was identified among human PPARδ mRNAs in this study. The majority of 5'-UTRs have exon 1 in the 5'-end, and reporter gene analysis using Huh7 and HeLa cells suggests that the main promoter of human PPARδ is positioned upstream of exon 1. As previously reported [25], this promoter does not have a consensus TATA-box but is rich in potential Sp-1 binding elements, a typical feature for house-keeping genes. This region contains evolutionary conserved areas between the human and mouse PPARδ genes. Alternative promoters could, however, be present in the human PPARδ gene, as is the case for the mouse orthologous gene [27]. Contrary to this assumption, reporter gene analysis using Huh7 and HeLa cells showed a low promoter activity related to only one of the four alternative promoter regions tested. Analysis of other cell types could, however, provide a different result. The identified alternative 5'-ends might represent tissue- or developmental-specific alternative transcription starts sites or be the products of as yet unknown post-transcriptional processing. A tissue-specific alternative transcription start site could apply to one alternative 5'-end, which was detected only in cDNA from pancreas.

Several levels of regulation of PPAR activity have been reported such as phosphorylation by kinases that affects the transactivation potential and ubiquitin-proteosome degradation, a mechanism to arrest transcriptional activation [26,34-38]. The post-transcriptional regulation by 5'-splicing suggested here for human PPARδ and previously reported for the mouse orthologous gene [27] could represent one additional level of regulation. At least eight exons can be included in the 5'-UTR region of human PPARδ transcripts by alternative splicing; the previously identified exons 1, 2 and 3 [25] and the five exons (2a-2e) identified in this study. The untranslated human PPARδ exons are not mutually exclusive but rather appear to be spliced-in forming a diversity of 5'-UTRs containing any combination of exons. In addition to the splice variants discussed and depicted in Figure 1B, ten more combinations involving the herein identified untranslated exons and the previously reported untranslated exons 1, 2 and 3 have been observed, either by sequencing of PCR products in this study or in data from the human EST-data base. The newly identified exons are positioned within the 63 kb long sequence separating exon 2 and the first coding exon. Interestingly, all PPAR isoforms share a similar gene organization with long introns separating untranslated exons from coding exons. The presence of several new splice variants affecting the 5'-UTR region of PPARγ was recently reported [39]. Likewise multiple isoforms of human PPARα with variable 5'-UTRs are presented in the NCBI HomoloGene database.

Different combinations of 5'-untranslated human PPARδ exons form 5'-UTRs of various length and numbers of upstream AUGs. The originally postulated scanning mechanism for initiation of translation predicts that translation should be initiated at the first AUG codon closest to the 5'-end of the mRNA [40]. However, upstream AUGs that are followed by a run of codons that ends in a termination codon within the 5'-UTR, so called upstream open reading frames (uORFs), play a role in translational regulation [41,42]. The strong correlation between the number of upstream AUGs and the observed translation efficiency indicates that the regulation of the yield of PPARδ per unit mRNA involves uORFs. An influence on translation by GC rich regions with a potential to form stable secondary structures in the more extended 5'-UTRs cannot be ruled out. The possibility of an initiation of translation by internal ribosomal entry sites (IRES) that circumvents upstream AUG codons and secondary structures is intriguing. The presence of IRES in the 5'-UTRs of many growth regulatory genes is believed to provide a means for the tuning of their translation in time and space and in response to relevant stimuli [43].

Our results and available human EST data suggest a ubiquitous occurrence of the newly identified exons in human PPARδ mRNAs. Quantification using real-time PCR, however, showed that the overall preferred 5'-UTR is short, containing only exons 1 and 2, thus representing the majority of the human PPARδ transcripts. Besides this, only mRNA isoforms containing exon 2a are present in measurable amounts. Considering that the presence of full-length exon 2a contributes substantially to the reduced translation efficiency, it is tempting to speculate that occurrence of exon 2a might be of importance for the functional roles of human PPARδ.

The generation rate of new exons has been calculated to approximately 2.71 × 10^-3 ^per gene per million years [44]. Most new exons are considered to originate from exonization of intronic sequences and appear in minor splice forms, which is in agreement with the finding in this study. An important question that remains to be answered is whether the low-abundant splice variants containing the newly identified exons represent biologically significant and functional isoforms. Comparative genomics was used as a guide to identify conserved sequence element, considering that functional regions generally are under strong selection pressure to stay unchanged. The previously identified untranslated exons 1 and 2 are highly conserved between species whereas the newly identified human PPARδ exons are either human- or primate-specific exons. Exon 2d was identified as a human-specific exon. All other novel exons are highly conserved in chimpanzee whereas only two exons (exons 2a and 2b) show the same high conservation in macaque. Likewise, all features related to the four alternative 5'-ends identified for the human PPARδ are fully conserved in chimpanzee whereas only one, the 5'-end related to exon 2a, is preserved in the macaque. At present, however, there is no available EST data to predict alternative splicing or promoter usage of PPARδ in chimpanzee or macaque.

The sequence of exon 2a appears to be the most conserved of the five new human PPARδ exons. All features related to this exon, including internal splice junctions and a putative 5'-end, are fully conserved in both the chimpanzee and macaque genes. Alternative splicing involving a homologous exon 2a in chimpanzee is suggested by available PPARδ isoforms for this species. The overall conservation of the exon 2a sequence is surprisingly high in the dog gene. However, it is highly unlikely that an orthologous exon is spliced in this species.

Taken together, it is likely that transcripts with a short 5'-UTR provide a constant basal level of PPARδ synthesis. Transcripts with extended 5'-UTRs, such as the mRNA isoforms containing full-length exon 2a, with multiple AUG codons and potential secondary structures, could provide a spatio-temporal regulation of protein expression during for example growth, development, differentiation and stress. It has been reported that dysregulation of key proteins regulating growth and differentiation via modulation of 5'-UTR activity plays a role in the development or progression of various forms of cancer [43]. Whether mechanisms related to alternative splicing of PPARδ could contribute to the conflicting findings regarding the role of this nuclear factor in cancer development remains to be investigated [13]. Likewise, the role of alternative promoters in the regulation of human PPARδ gene expression is a matter to consider in future studies.

The possibility of a dominant negative variant of the human PPARδ adds to the complexity of PPARδ as a transcriptional regulator. The alternative polyadenylation signal and premature stop-codon in human PPARδ intron 8 are not conserved in mouse, indicating that this isoform is human-specific, similar to the dominant negative variants of PPARα and PPARγ [45,46]. Suggested orthologs of the human PPARδ2 in chimpanzee and macaque, on the other hand, indicate that this could be a primate-specific isoform. Investigation of 5'-UTRs among human PPARδ2 transcripts shows that this isoform, similar to full-length PPARδ, is preferentially transcribed from a promoter upstream of exon 1. Since the variability among 5'-UTRs of PPARδ2 is less pronounced, this isoform appears to be less subjected to alternative splicing and thus translational regulation.

Transcripts of PPARδ2 were identified in placenta and adipose tissue. The occurrence and functional implications of PPARδ2 transcripts translated into protein in different cells and tissues, however, remains to be elucidated. Here we have shown for the first time *in vitro *that the isoform PPARδ2 represses the *trans-*activating ability of full-length PPARδ. The mode of action of dominant negative nuclear receptors in general is either competition for binding of DNA, auxiliary factors and cofactors or an effect on subcellular localization. A dominant negative mutant of the full-length mouse orthologous of PPARδ (E114P) has been tested in a similar setting with a PPRE reporter gene construct and GW501516, and shown to dose-dependently inhibit PPARδ induced luciferase activity. Contrary to PPARδ2 this full-length mutant is suggested to bind ligand and PPRE but remains in a repressive form and acts by competing with wild type PPARδ for binding to DNA [47,48]. The result of the mobility shift assay presented here indicates that PPARδ2 exerts its inhibitory properties by another mechanism than competition for binding to PPREs.

## Conclusion

The main finding of this study is the demonstration of multiple 5'-UTR splice variants of PPARδ mRNA that can influence the translation capacity. Moreover, the alternative splicing of human PPARδ is also reflected by the generation of a dominant negative isoform. Considering that PPARδ is widely expressed, a master regulator of gene transcription and acts as a metabolic sensor, proper understanding of the factors affecting the expression level and activity of this nuclear receptor is of crucial importance. Dysregulation of alternative splicing might promote human disease [49]. In the case of human PPARδ, this would entail dysregulation of protein expression with effects on both the *trans-*activating and repressor potentials of PPARδ.

## Methods

### Oligonucleotides and TaqMan probes

Oligonucleotides used in this paper were purchased from Proligo (France) and Thermo Hybaid (Germany). The sequences of RACE and PCR primers are listed in Table 5 and TaqMan primers and probes are listed in Table 3.

**Table 5:** Oligonucleotide primers used for RACE and PCR in this study

**Name**	**Target**	**Sequence (5'-3')**
Forward primers		
Ex1:1Fw	exon 1	5'-TTAAGCTTGGTACCCGCGGACCTGGGGATTAATGG
Ex1:2 Fw	exon 1	5'-AATTCTGCGGAGCCTGCGG
Ex2Fw	exon 2	5'-ACGTGATACTCACACAGTGG
Ex2a:1Fw	exon 2a	5'-GGAGCTCCAGGGAATACAATGTTCA
Ex2a:2Fw	exon 2a	5'-CAGGCCCAGTGGCAGCAAT
Ex2cFw	exon 2c	5'-GGCACTTGATGCTGTTGAATGGAG
Ex2eFw	exon 2e	5'-CCAGCTCTCCTTGACCTTGAACCTT
Ex3Fw	exon 3	5'-AACTGAAGCCCGTGGAGCA
Ex4Fw	exon 4	5'-GACCACAGCATGCACTTCCT
Ex8:1Fw	exon 8	5'-AGTGGCTTTGTCACCCGTGAGTTCCTG
Ex8:2Fw	exon 8	5'-TCCGCAAACCCTTCAGTGATA
In8Fw	intron 8 *	5'-GGCTGAGCCAGGAGAATCGCTTGA
Reverse primers		
Ex2Rev	exon 2	5'-CTCGTTGGTGCATCTGTCTTCA
Ex2a:1Rev	exon 2a	5'-CGCTCAGGAGGTACTTTGACCTCTTCC
Ex2a:2Rev	exon 2a	5'-GCTCAGGGATGAACTGGACAACTGAC
Ex2cRev	exon 2c	5'-ATGCTTCTGCTCAGAACCCAGCTCTCA
Ex2eRev	exon 2e	5'-AAGAGTGAAGGGCAGCTGAGTCCTGA
Ex3Rev	exon 3	5'-GAAGCAGTCCTGTAGAGATCAT
Ex4:1Rev	exon 4	5'-CCCAGTCATAGCTCTGGCATCGTCT
Ex4:2Rev	exon 4	5'-TGGCTGCTCCATGGCTGATCT
Ex5Rev	exon 5	5'-GTCACAGCCCATCTGCAGTT
Ex6Rev	exon 6	5'-CACTTCTCGTACTCCAGCTTC
Ex7Rev	exon 7	5'-TGCCACCAGCTTCCTCTTC
Ex8Rev	exon 8	5'-CCAGCCCCTTCTCTGCCT
Ex9:1Rev	exon 9	5'-CTGGAGCAGAGGGTGCAGCGAGGTCT
Ex9:2Rev	exon 9	5'-ATGGCATGCCCAGCTGTGC
In8:1Rev	intron 8*	5'-GATTACAGGTGTGAGCCACCACGTCTG
In8:2Rev	intron 8*	5'-CCCACATTAACATGACAGCAT

The primers are annotated according to the target exon or intron in the human PPARδ gene.

* Comparable to the 3'-UTR of PPARδ2

### Rapid amplification of cDNA ends (RACE)

Marathon-Ready cDNAs (Clontech) from human placenta, pancreas and adipose tissue were utilized for RACE studies according to the manufacturer's instructions. Two sequential rounds of 5'-RACE were carried out with the reverse gene-specific primer (GSP) Ex7Rev, followed by Ex6Rev or Ex3Rev in combination with adapter primers (AP1 and AP-2; Clontech). Purified fragments were sequenced using Big Dye Terminator Kit and an automatic sequencer (Genetic Analyzer 3100, Applied Biosystems). Nested PCR was subsequently performed using primers Ex5Rev, Ex4:1Rev, Ex2a:1Rev, Ex2a:2Rev, Ex2cRev and Ex2eRev combined with the adapter primer AP2. Sequencing was performed either directly on the PCR products or on samples of gel-purified fragments when multiple bands were obtained.

Another round of 5'-RACE was conducted on Marathon-Ready cDNAs using either a full-length PPARδ (PPARδ1) primer (Ex9:1Rev) or a PPARδ2 specific primer (In8:1Rev) in combination with the adapter primer AP1. Further rounds of PCR on these 5'-RACE products, thus enriched in either PPARδ1 or PPARδ2, were performed using exon/exon specific primer pairs specified in the Result section (Figure 5).

Two sequential rounds of 3'-RACE were carried out using Marathon-Ready cDNAs and the gene-specific forward primers Ex8:1Fw and In8Fw in combination with adapter primers AP1 and AP-2, respectively. Sequencing was performed on PCR products as described above.

### RNA extraction, reverse transcription, PCR and real-time PCR analysis

Total RNA was extracted using RNeasy system (Qiagen) from a panel of human cell lines; the human hepatocellular carcinoma cell lines HepG2 and Huh7, the cervical epithelioid carcinoma cell line HeLa, the human acute monocytic leukaemia cell line THP-1 and kidney epithelial carcinoma cell line A498. One microgram of total RNA was reverse transcribed with a poly-dT primer using Superscript II (Invitrogen) according to the manufacturer's instructions. First strand cDNAs were subjected to PCR using exon specific primer pair combinations (Table 5). The PCR cycles were as follows: 94°C for 3 min followed by 36 cycles of 94°C for 20 sec, 55°C for 30 sec, 72°C for 30 sec, and finally 72°C for 10 min. Sequencing was performed on PCR products as described above. Real-time PCR (TaqMan analysis) was performed on cDNA from human cell lines and tissues according to the manufacturer's instructions (Applied Biosystem, USA) and the reaction conditions involved denaturation for 10 min at 95°C, and 45 cycles of amplification with 15 sec at 95°C and 1 min at 60°C. Primers and probes are listed in Table 3. Two different cDNAs obtained using total RNA from each cell line (described above) or tissue (Clontech) were analysed twice and determined in triplicates using an ABI prism 7000 (Applied Biosystem). Standard curves were run for all assays to ensure consistent amplification efficacy. All variants were normalised to the Ex8:9 assay using the comparative C_T_-method (User Bulletin #2, December 11, 1997 (updated 10/2001); ABI PRISM 7700 Sequence Detection System) and presented as relative expression levels.

### Reporter gene constructs, transient transfections and luciferase assays

A luciferase reporter gene construct covering the proximal promoter region of the human PPARδ gene upstream of exon 1, from -733 to + 44 cloned into pGL3 Basic (Promega) (hereafter referred to as the -733 bp construct) has previously been described [50]. Additional constructs covering longer (2.6 kb and 1.5 kb) and shorter sequences (171 bp, 61 bp and 48 bp) of this promoter were created from restriction enzyme digests of a genomic PPARδ clone [25] and the -733 bp construct. Likewise, genomic upstream sequences (approximately 1 kb and 250 bp) for each of the four alternative transcription start sites identified in this study were PCR amplified and cloned into pGL3 Basic.

The Huh7 and HeLa cell lines were maintained in Dulbecco's modified Eagle's medium (D-MEM 1g/ml glucose, Invitrogen) supplemented with 10% fetal bovine serum, penicillin (100 units/ml) and streptomycin (100 ug/ml) at 37°C in 5% CO_2 _in air. Approximately 1 × 10^5 ^cells/well were plated on a 24-well plate and transiently transfected after 24 h with 100 ng of the reporter gene constructs or empty vector using Lipofectamin (Invitrogen) according to the manufacturer's protocol. Plasmid pSV-β-galactosidase control vector (Promega) (260 ng) was co-transfected for normalization of the transfection efficiency. After 24 h, cells were washed, lysed and luciferase activity was measured as previously described [51]. The β-galactosidase enzyme assay system (Promega) was used for measuring the β-galactosidase activity in cell lysates according to the manufacturer's instructions. The activities were determined in quadruplicates and the data presented are based on the mean (± SD) luciferase/β-galactosidase ratios of three independent transfections, setting the value of empty vector to 1.

### Coupled transcription/translation

A fragment covering the full-length PPARδ mRNA sequence from exon 1 to the 3'-UTR was amplified using Marathon placenta cDNA and primers Ex1:1Fw (introducing a KpnI restriction site in the 5'-end) and Ex9:2Rev. The fragment was cloned into the pTNT vector (Promega) using the restriction sites KpnI and SmaI in the vector and KpnI and MscI in PPARδ, respectively. Variable 5'-UTRs upstream of exon 4 were introduced using the primer- based KpnI site in the 5'-end of exon 1 and an NcoI site in exon 4. The 5'-UTR sequences were amplified using Marathon cDNAs and combinations of exon specific primers Ex1:1Fw, Ex2a:1Fw, Ex2a:2Fw, Ex2eFw, Ex2eRev, Ex3Rev and Ex4:2Rev. Longer 5'-UTRs were amplified in two pieces, subcloned in PGEM-T vector (Promega) and ligated together prior to being introduced in the 5'-end of PPARδ in pTNT vector. Coupled *in vitro *transcription and translation was performed on these plamids using a TNT Quick Coupled Transcription/Translation System (Promega) and [^35^S]methionine (Amersham) according to the manufacturer's instructions. The [^35^S]methionine labelled products were separated by 10% SDS-polyacrylamide gel electrophoresis (SDS-PAGE), subjected to autoradiography and analysed on a PhosphorImager scanner (Fuji film Bas-2500, Australia). The *in vitro *translated products were also subjected to Western blot analysis under reducing conditions on 10% SDS-PAGE as described by Laemmli [52] using the polyclonal PPARδ specific antibody IMG-3297 (Nordic Biosite, Sweden) and a horseradish peroxidase-labelled secondary antibody. Detection was performed with the ECL Advanced Western blot detection system (Amersham). Both the autoradiographic and Western blot bands were quantified using the NIH ImageJ software [53]. The amounts of plasmids used (1 μg) were within a range of saturating conditions where the outcome of the transcription/translation reactions was independent on the differences in target DNA concentration introduced by the different lengths of the 5'-UTRs.

### Expression vectors for PPARδ, transient transfections and Luciferase assays

The expression vector for full-length PPARδ (PPARδ1) under the control of the human cytomegalovirus (CMV) promoter was a kind gift of Dr CN Palmer and is described elsewhere [54]. The expression vector for PPARδ2 was created using an EcoRV site in the coding region, common to both PPARδ1 and 2, and replacing the downstream sequence of the expression vector with the corresponding 3'-sequence of PPARδ2. The 3'-sequence of PPARδ2 was obtained by PCR using a genomic clone of PPARδ as template [25], and the forward primer Ex8:2Fw and reverse primer In8:2Rev. The reporter construct pFABPLUC containing four copies of the PPRE from the human liver FABP gene in front of the HSV-TK promoter is described elsewhere [54]. Growth and transfection of HeLa cells, Luciferase assay, and β-galactosidase assay were performed as described above. Nuclear extract prepared as previously described [55] from HeLa cells transfected with the expression vectors for PPARδ1 and PPARδ2, respectively, was subjected to Western blot analysis as described above, using the PPARδ antibody sc-7197 (Santa Cruz Biotechnology). Nuclear extract prepared from untransfected HeLa cells was used as a negative control.

### Electrophoretic mobility shift assay, EMSA

The binding properties of PPARδ1 and PPARδ2, respectively, to the functional PPRE of the rat acyl-CoA oxidase gene with and without the heterodimer partner RXR were investigated by EMSA. Human PPARδ1 and PPARδ2, cloned into the TNT vector, and TNT-hRXRα (a kind gift from Dr Glinghammar, AZ, Sweden) were *in vitro *transcribed/translated using the rabbit reticulocyte lysate system (Promega) as previously described but with unlabelled methionine. Incubation for EMSA was conducted as previously described [56] using 6 μl of *in vitro *produced PPARδ1 or PPARδ2 with 2 μl of RXRα or mock lysate produced using an empty vector, in a total volume of 20 μl (2 μg poly(dI-dC), 0.75 mM EDTA pH 8.0, 18 mM HEPES pH 7.9, 0.5 mM dithiothreitol, 4% Ficoll), and [γ-^32^P]-end labelled double stranded ACO-oligo (5'-ggaccAGGACAaAGGTCAcgttcgg-3')[57]. For competition, a 100-fold molar excess of unlabelled double stranded ACO-oligo was added. A PPARδ antibody sc-7197 was used for supershift. This antibody, directed against the N-terminal region (epitope 2–75) of human PPARδ, was shown to detect both variants of *in vitro *translated PPARδ using Western blot (data not shown). DNA-protein complexes were applied to a 6% polyacrylamide gel and run for 4 hours at 200V in 0.25 × TBE at 4°C. The gel was dried, subjected to autoradiography and analysed on a PhosphoImager scanner.

### Bioinformatics and comparative genomics

The NCBI human Genome Browser, the human EST-search tool and homologue RefSeq of PPARδ were provided by NCBI [58]. The Evolutionary Conserved Regions Browser tool (ECR Browser) [59] was used to identify sequence conservation between species, ClustalW [60] for sequence alignments and sequence identity scores and the RepeatMasker [61] to unravel repetitive elements in PPARδ genes. Localization and extraction of relevant sequences for comparative analysis between species was performed using the ECR Browser tool and defined regions were subsequently extracted from the PPARδ gene RefSeq for each species and aligned to the relevant human exon sequences using ClustalW.

## Abbreviations

PPAR, peroxisome proliferator-activated receptor; PPRE, peroxisome proliferator response element; RACE, rapid amplification of cDNA ends; GSP, gene specific primer; UTR, untranslated region; RT, reverse transcription; PCR, polymerase chain reaction; SDS, sodium dodecyl sulfate; bp, base pair(s); ORF, open reading frame; uORF, upstream open reading frame; EST, expressed sequence tags; RefSeq, reference sequence

## Authors' contributions

KL performed all RACE, RT-PCR analysis, reporter gene assays, bioinformatics and comparative genomics including analysis and interpretations and drafted the manuscript. PT performed all in vitro coupled transcription/translation, Western Blot, EMSA and Real-time PCR experiments including data analysis and interpretations and co-authored the manuscript. EE coordinated the study and co-authored the manuscript and was responsible together with KL for the study design. AH co-authored the manuscript and was involved in project development. All contributing authors reviewed and approved the final copy of this manuscript.
